# Using GRACE satellite observations for separating meteorological variability from anthropogenic impacts on water availability

**DOI:** 10.1038/s41598-020-71837-7

**Published:** 2020-09-15

**Authors:** Seyed-Mohammad Hosseini-Moghari, Shahab Araghinejad, Kumars Ebrahimi, Qiuhong Tang, Amir AghaKouchak

**Affiliations:** 1 Key Laboratory of Water Cycle and Related Land Surface Processes, Institute of Geographic Sciences and Natural Resources Research, Chinese Academy of Sciences, Beijing, 100101 China; 2 Stantec Consulting Company, Sacramento, CA 95816 USA; 3 Department of Irrigation and Reclamation Engineering, University of Tehran, 14378-35693 Karaj, Iran; 4 University of Chinese Academy of Sciences, Beijing, 100049 China; 5 Department of Civil and Environmental Engineering, University of California, Irvine, 92697 USA; 6 Department of Earth System Science, University of California, Irvine, 92697 USA

**Keywords:** Hydrology, Hydrology

## Abstract

Gravity Recovery and Climate Experiment (GRACE) observations provide information on Total Water Storage Anomaly (TWSA) which is a key variable for drought monitoring and assessment. The so-called Total Water Storage Deficit Index (TWSDI) based on GRACE data has been widely used for characterizing drought events. Here we show that the commonly used TWSDI approach often exhibits significant inconsistencies with meteorological conditions, primarily upon presence of a trend in observations due to anthropogenic water use. In this study, we propose a modified version of TWSDI (termed, MTWSDI) that decomposes the anthropogenic and climatic-driven components of GRACE observations. We applied our approach for drought monitoring over the Ganges–Brahmaputra in India and Markazi basins in Iran. Results show that the newly developed MTWSDI exhibits consistency with meteorological drought indices in both basins. We also propose a deficit-based method for drought monitoring and recovery assessment using GRACE observations, providing useful information about volume of deficit, and minimum and average time for drought recovery. According to the deficit thresholds, water deficits caused by anthropogenic impacts every year in the Ganges–Brahmaputra basin and Markazi basins is almost equal to an abnormally dry condition and a moderate drought condition, receptively. It indicates that unsustainable human water use have led to a form of perpetual and accelerated anthropogenic drought in these basins. Continuation of this trend would deplete the basin and cause significant socio-economic challenges.

## Introduction

Drought is a multifaceted natural phenomenon that occurs across a wide range of spatio-temporal scales^1^ with adverse environmental, social and economic impacts^2, 3^. Drought often leads to reduction in agricultural production^4, 5^, job losses^6, 7^, more frequent dust storms^8, 9^, more intense forest fires^9, 10^, and many other negative impacts^11–13^. For instance, in the first half of 2001, drought caused $2.6 billion economic loss in Iran^14^. In 2014, California drought caused $2.2 billion economic loss and 17,100 job losses^6^. Also, 25–45% reduction in crop yield across Australia during the millennium drought periods has been reported^4^. On the other hand, global warming and climate change is expected to increase the frequency and intensity of droughts^15–18^. In today's world with limited water resources and ever increasing water demands, impacts of drought can become more intense as a result of population growth and more competition for water; a phenomenon termed anthropogenic drought^19^. Therefore, developing drought monitoring and predictions are fundamental to reduce drought impacts.

In recent decades, drought monitoring has improved substantially using satellite vegetation health products^20–22^, ground-based and satellite precipitation observations^23–25^, soil moisture data^26–28^, and numerical model simulations^28–30^. However, performance of these products and numerical models needs to be verified for operational application. Different datasets and monitoring systems have their own advantages and limitations and they do not capture all aspects of droughts, particularly groundwater and total water storage^31^. After launching Gravity Recovery and Climate Experiment (GRACE) satellite in March 2002, a unique dataset on Total Water Storage Anomaly (TWSA) has become available worldwide^32–34^. GRACE data has been evaluated in a variety of studies and has also been used as a benchmark and/or input for evaluating or improving hydrological/land surface model simulations^32, 35–39^. Owing to uniqueness and global coverage of GRACE observations, it has been widely used in drought monitoring studies^38, 40–49^.

Although GRACE provides valuable data for drought monitoring worldwide, its application in heavy irrigated/managed regions, that are often more vulnerable to droughts, is not straightforward. GRACE observations include not only climatic information, but also human factors^50–53^. In regions with intensive human activities, for example, GRACE signals may be affected by human induced mass changes^54, 55^. In areas where water consumption is higher than replenishment rate, GRACE observations typically indicate a decreasing trend. While this information is valuable and provides insights on anthropogenic water use, it poses a challenge specially where we observed a significant downward trend: When standardizing TWSA time series, at the start, middle and end years of time series, we typically observe wet, near normal and dry years, respectively, primarily dominated by a downward trend^56^. As a result, as new observations are recorded, a past drought event may be classified into a normal or even wet period after re-standardization of TWSA data. For instance, monitoring drought over the Tigris-Euphrates basin using GRACE observations, Chao et al.^57^ stated that the Tigris-Euphrates basin underwent a severe drought from September 2007 to end of 2015. This conclusion appears to be inconsistent with the apparently wet climate of the region in that period. It seems that this TWSA-based conclusion is dominated by an overall downward trend in TWSA in the region. To overcome this limitation, using a signal decomposition approach, Hosseini-Moghari et al.^56^ proposed a modified GRACE-based drought index called modified total storage deficit index (MTSDI), designed to obtain drought information consistent with meteorological variability even when there is a significant trend in the data. Inspired by their approach, this paper introduces a modified drought index based on Zhao et al.^41^ termed modified total water storage deficit index (MTWSDI). Unlike the previously introduced MTSDI, MTWSDI can be used to differentiate between meteorological droughts and anthropogenic droughts using GRACE observations. Additionally, this study defines a threshold-based drought monitoring and recovery framework using GRACE observations, specifically designed for drought management, early warning, and recovery assessment.

**Figure 1:**
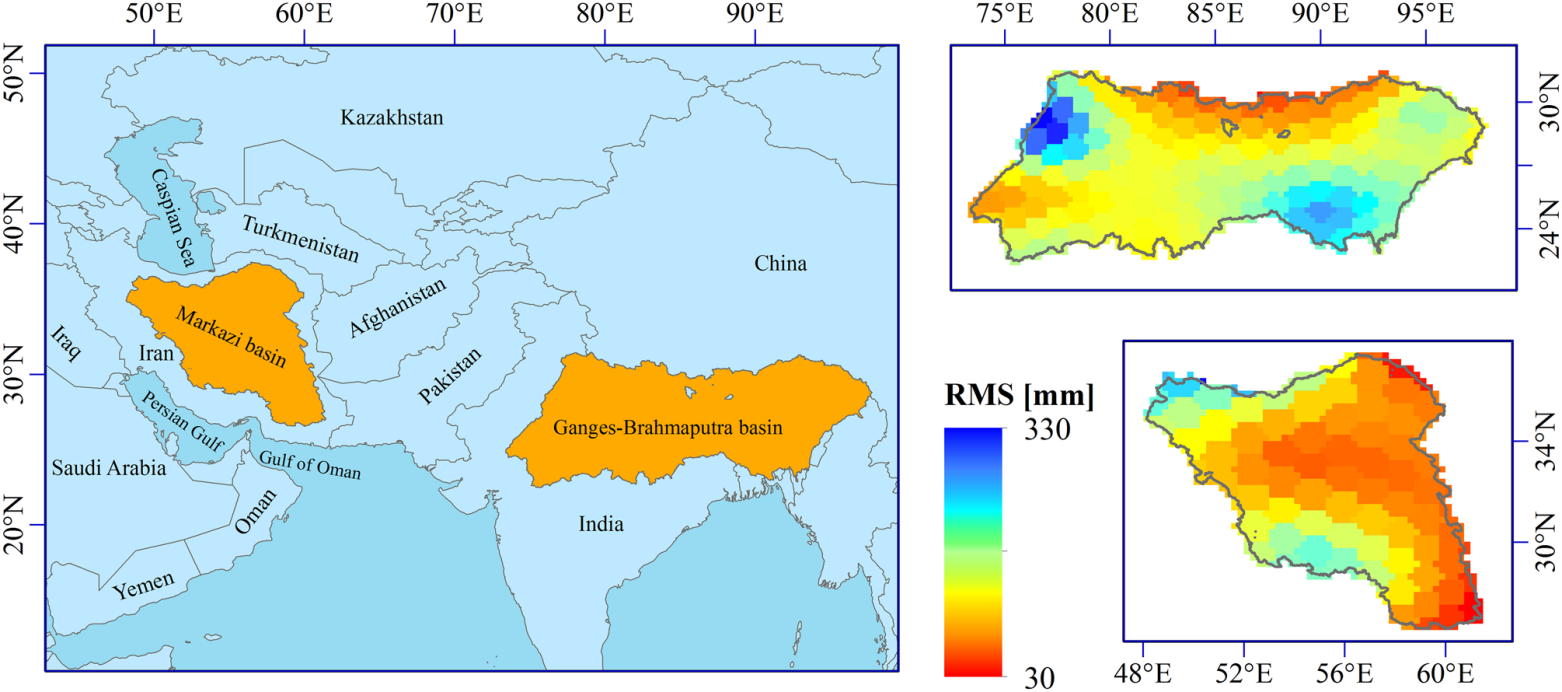
Location of the studied basins (left) and the root mean square (RMS) of TWSA over the basins (right).

We carried out drought monitoring over two irrigated basins in humid and arid climates (see Fig. 1). The selected study areas are two basins with significant water consumptions. The Ganges–Brahmaputra basin located in South Asia in humid climate with an area of 1,689,100 km^2^ and 21.26% irrigated area^52^. This basin is located within India (more than 60%), China, Nepal, Bangladesh and Bhutan. Ganges and Brahmaputra receive annually 1,550 and 2025 mm precipitation, respectively^58^. Life of over half a billion people in rural areas within the basin relies on agriculture and availability of freshwater^59^. The second basin is the Markazi basin in Iran, located in arid climate with an area of 844,356 km^2^ and annual precipitation of 165 mm. The Markazi basin receives 29% of the total renewable water resources of Iran^60^, while most of the population live in this area. In addition, in the rural area of the basin, agriculture is the regular job where most groundwater is used to meet the irrigation needs. Considering that the occupation of many people in two basins is linked to water, drought impacts are more intense in these areas.

## Results

### Assessing TWSA time series

**Figure 2:**
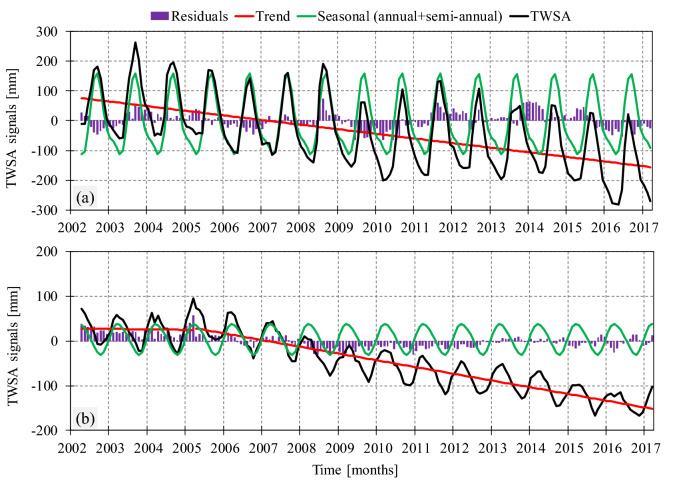
TWSA time series and its components over (**a**) the Ganges–Brahmaputra basin and (**b**) the Markazi basin. TWSA time series decomposed into long-term trend (red lines), annual and semi-annual amplitudes (green lines) and residues (purple columns). Residues were calculated by subtracting the long-term trend and annual and semi-annual terms from TWSA.

Figure 2 shows TWSA time series and its components including the trend, seasonality, and residuals (see “Data and methods” sections). In time series of the Ganges–Brahmaputra basin, a decreasing trend exists (Fig. 2a, red line), which is significant at 95% confidence level based on the Mann–Kendall trend test. This trend indicates annual loss of − 15.48 mm of water storage over the basin. For the Markazi basin (Fig. 2b), in the first 3 years, there exists an insignificant increasing trend, and afterwards a significant decreasing trend with the loss rate of − 15.05 mm/year. A continuous negative trend in both time series is evidence of the anthropogenic effect on TWSA over the basins. Figure 2c,d illustrate residuals of TWSA time series of the Ganges–Brahmaputra and Markazi basins, respectively. In our study, we decompose TWSA to systematic (e.g., possible trends, annual variability and seasonal changes) and stochastic terms (here, residuals)—See “Data and methods” section. Residual is defined as the difference between the TWSA and the sum of other systematic terms. We then use normalized residuals to describe meteorological droughts in the selected basins. In Fig. 2, continuous negative values of residuals indicate drought events. We have developed the Total Water Storage Deficit Index (TWSDI) and Modified Total Water Storage Deficit Index (MTWSDI) based on TWSA and its residuals time series, respectively. Furthermore, time series of the Standardized Precipitation Index (SPI) and Standardized Precipitation Evapotranspiration Index (SPEI) on different time scales have been calculated over two studied basins as a benchmark for meteorological variability. Table 1 illustrates the drought categorization based on the selected indices, ranging from abnormal dry (i.e., a standardized index value less than − 0.5) to exceptional drought (i.e., a standardized index value less than − 2).

**Table 1:** Drought categories information based on standardized drought indices and their corresponding D-scale.

Standardized index	Category	D-scale
− 0.50 to − 0.79	Abnormally dry	D0
− 0.80 to − 1.29	Moderate drought	D1
− 1.30 to − 1.59	Severe drought	D2
− 1.60 to − 1.99	Extreme drought	D3
− 2.0 or less	Exceptional drought	D4

### Correlations between GRACE-based drought indices and metrological drought indices

Table 2 shows the Correlation Coefficients (CCs) between GRACE-based drought indices and SPI/SPEI. Based on the results, in the Ganges–Brahmaputra basin, the commonly used TWSDI is poorly correlated with SPI and SPEI. CCs between TWSDI and SPI/SPEI are not statistically significant at any time scale (at 0.05 significance level). This is a major limitation of the existing methods. Meanwhile, CCs between our proposed index (i.e., MTWSDI) and SPI/SPEI on a 12-month scale reach 0.57 and 0.61, respectively. All CCs are significant on all the time scales (at 0.05 significance level) except the 1-month SPI. Note that droughts are often defined at longer than 1-month time scale to ensure persistence of dryness. For the Markazi basin, although TWSDI is weakly correlated with the metrological drought indices on long timescales, MTWSDI still outperformed TWSDI. The highest CC value was obtained between MTWSDI and SPI/SPEI on a 12-month time scale, being equal to 0.62 and 0.51, respectively. Based on the results, GRACE-based drought indices may not be appropriate for detecting meteorological droughts at short-time scales (e.g., 1- to 3-month SPI and SPEI). However, MTWSDI can be efficiently used for drought monitoring at longer time scales. This highlights that GRACE-based drought analysis often contradicts the meteorological condition primarily due to trends in the data. The proposed method leads to an expected consistency between anthropogenic drought (based on GRACE) and meteorological droughts, yet allows differentiating between the two.

**Table 2:** Correlation coefficients between GRACE based drought indices and metrological drought indices on a different time scale.

Indices	Ganges–Brahmaputra		Markazi	
	TWSDI	MTWSDI	TWSDI	MTWSDI
SPI 1	0.05	0.13	− 0.05	0.13
SPI 3	0.13	**0.31**	0.00	**0.33**
SPI 6	0.12	**0.40**	**0.16**	**0.49**
SPI 9	0.06	**0.48**	**0.26**	**0.57**
SPI 12	0.07	**0.57**	**0.31**	**0.62**
SPI 24	0.04	**0.54**	**0.42**	**0.61**
SPI 36	0.01	**0.50**	**0.29**	**0.39**
SPEI 1	0.05	**0.17**	0.10	0.11
SPEI 3	0.12	**0.37**	**0.19**	**0.27**
SPEI 6	0.11	**0.49**	**0.27**	**0.39**
SPEI 9	0.05	**0.56**	**0.32**	**0.45**
SPEI 12	0.06	**0.61**	**0.35**	**0.51**
SPEI 24	− 0.01	**0.59**	**0.43**	**0.46**
SPEI 36	− 0.02	**0.50**	**0.25**	**0.24**

Bold values indicate significance < 0.05 based on *t-*test.

**Figure 3:**
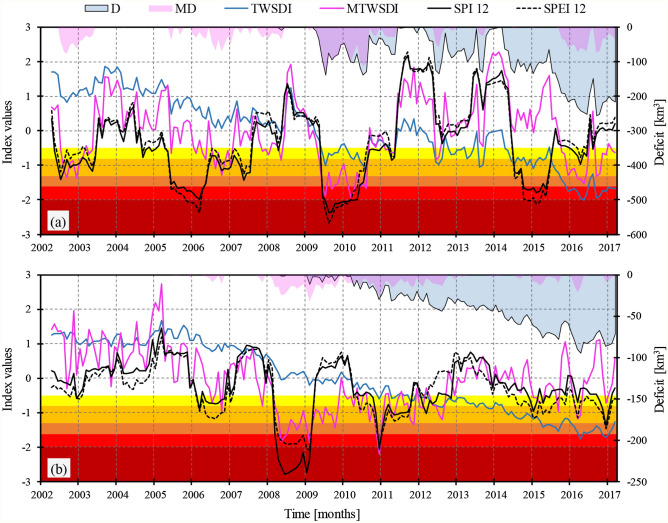
Time series of GRACE-based drought indices (TWSDI and MTWSDI), metrological drought indices (SPI and SPEI), deficit (D) and modified deficit (MD) over (**a**) the Ganges–Brahmaputra basin and (**b**) the Markazi basin.

Considering the fact that CCs between GRACE-based drought indices and 12-month SPI/SPEI were the largest, we have compared these time series in Fig. 3 for more detailed analysis. As shown in Fig. 3a, in the Ganges–Brahmaputra basin, although TWSDI during 2009–2010 has the same behaviour as the meteorological indicators (SPI and SPEI), it highly underestimates drought severity. Generally, TWSDI time series follow the trend in the original TWSA (here, a negative trend). MTWSDI, on the other hand, corresponds well with the SPI and SPEI behaviour. However, in some cases there is a lag between detected drought events. For example, the meteorological drought event between 2014 and 2015 was translated to a drought event in 2015–2016 based on MTWSDI. This showed the storage changes in the basin may occur after some months. In the Markazi basin, TWSDI exhibits a decreasing trend that does not correspond to meteorological droughts, and is likely the result of anthropogenic impact such as extensive overexploitation of groundwater^57, 61^. Therefore, TWSDI alone may not be suitable for describing droughts based on both meteorological and anthropogenic perspectives, at least in places with a substantial downward or upward trend. However, investigating both TWSDI and MTWSDI offers the opportunity to decompose the human and the climate signals. Figure 3 also shows drought deficits based on the widely used TWSDI (Deficits, D) and the proposed MTWSDI (Medfield Deficits, MD). As shown, D time series indicate a cumulative increasing pattern showing the combined effect of climate and human activities. Meanwhile, MD time series almost coincides with droughts recognized by SPI12 and SPEI12 and allows assessing the fraction of deficit that corresponds to meteorological drought events (see Fig. 3).

### Assessing drought using GRACE-based drought indices

For further evaluations, we have assessed drought events detected by TWSDI and MTWSDI over both basins. A drought event was considered as a period in which the standardized drought index remained less than − 0.5 for at least four continuous months. Based on TWSDI, three drought events were detected over the Ganges–Brahmaputra basin during 2002–2017; all drought events occurred after April 2009 (see Table 3). The first drought occurred from April 2009 to Aguste 2010 with a total severity of − 1,445 km^3^ months (see “Data and methods” section for more information)*,* which coincided with a meteorological drought based on SPI and SPEI (see Fig. 3). This is an example in which both the meteorological and GRACE-based information are consistent. The second drought event spans from April 2012 to November 2013 which is not consistent with SPI and SPEI results. The last one is a long-term 26-month drought event from April 2014 till the end of the study period. The latter event is consistent with the meteorological conditions between April 2014 and July 2015; however, it is not consistent with the meteorological conditions between August 2015 and March 2017. This suggests that the GRACE signal mainly shows the anthropogenic impacts on local water availability rather than meteorological drought in the river basin. Based on our proposed approach (i.e., MTWSDI), five drought events were recognized over the Ganges–Brahmaputra basin during the study period. The longest one spans from April 2009 to August 2010 with a total severity of − 1,315 km^3^ months. All drought events based on MTWSDI coincided with meteorological droughts. For the Markazi basin, only one drought event was detected by the commonly used TWSDI from April 2011 to end of the study period in which the basin lost about 57.75 km^3^ water on average each month (total severity of − 3,726 km^3^ months). However, this event is not consistent with meteorological information (Fig. 3). Meanwhile, MTWSDI identifies two drought events, both consistent with meteorological (SPI and SPEI) information (Fig. 3). The most severe drought is a 21-month event from March 2008 to November 2009 with a total severity of − 341 km^3^ months (see Table 3). The results show that the proposed MTWSDI performs better than the commonly used TWSDI in term of being consistent with meteorological condition. That is because TWSDI represents the combined impacts of both meteorological drought and anthropogenic activities on water availability, while MTWSDI represents impacts of meteorological drought on relatively short time scale. By comparing MTWSDI with TWSDI, the proposed method allows decomposing the impacts of meteorological drought and anthropogenic activities on water availability for more informed drought assessment. It should be noted that the de-trending in MTWSDI would inevitably remove impacts of meteorological drought at long time scale (36 months). Thus, the decomposed anthropogenic impacts may contain leaked signal from long term meteorological trend. However, the SPI data in the two basins show little long term trend, suggesting impact from long term meteorological trend is minor.

**Table 3:** Detected drought events based on TWSDI and MTWSDI from 2002 to 2017 (at least four continuous months with TWSDI/MTWSDI less than − 0.5 were considered a drought event).

Basin	TWSDI				MTWSDI			
	Time period	Duration (months)	Total severity (km^3^ months)	Coincides with a meteorological drought?	Time period	Duration (months)	Total severity (km^3^ months)	Coincides with a meteorological drought?
Ganges–Brahmaputra	Apr-09 to Aug-10	17	− 1,445	Yes	Jul-02 to May-03	11	− 495	Yes
	Apr-12 to Nov-13	20	− 1,437	No	Jul-06 to Feb-07	8	− 345	Yes
	Apr-14 to Jul-15	16	− 1,670	Yes	Apr-09 to Aug-10	17	− 1,315	Yes
	Aug-15 to Mar-17	20	− 4,198	No	Oct-15 to Jun-16	9	− 494	Yes
					Sep-16 to Mar-17	19	− 282	Yes
Markazi	Apr-11 to Mar-17	72	− 3,726	No	Mar-08 to Nov-09	21	− 341	Yes
					Jun-10 to Sep-12	28	− 291	Yes

### GRACE-based thresholds for drought monitoring

**Figure 4:**
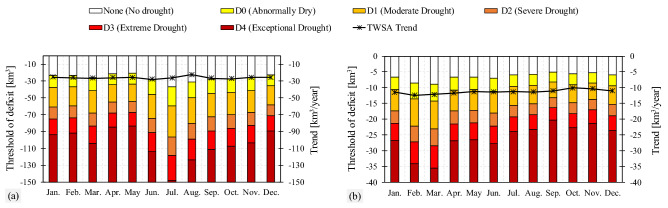
Thresholds of deficit related to each drought category in each month for (**a**) the Ganges–Brahmaputra basin and (**b**) the Markazi basin. The TWSA trend of each month during 2002–2017 is plotted.

As shown, in the previous sections, MTWSDI outperformed TWSDI for describing events consistent with meteorological conditions. Therefore, in the rest of this paper, we investigate the possibility to monitor meteorological drought solely based on MTWSDI. Figure 4 illustrates thresholds of deficit corresponding to each drought category and month (see “Data and methods” section). For instance, in January at the Ganges–Brahmaputra basin (Fig. 4a), an abnormally dry situation will start with a deficit of − 23.46 km^3^, a moderate drought results from a deficit between [− 37.57, − 61.05] km^3^. A deficit ranging [− 61.05, − 75.14] km^3^ indicates a severe drought, whereas a deficit of [− 75.14, − 93.93] km^3^ corresponds to an extreme drought. An event would be considered an exceptional drought if the absolute value of deficit exceeds − 93.93 km^3^ in January. For the Markazi basin, due to its arid climate, the absolute value of drought threshold is less than the Ganges–Brahmaputra basin. For example, in January, an abnormally dry situation corresponds to a deficit between − 6.65 and − 10.64 km^3^. In addition, moderate, severe, extreme and exceptional drought deficit thresholds are − 10.64, − 17.29, − 21.28 and − 26.60 km^3^, respectively. These thresholds can be calculated for different regions around the world to develop a deficit-based drought monitoring system using GRACE observations. The monthly trend of TWSA is also plotted in Fig. 4 (see the black lines). The trend of TWSA is negative over all the months, indicating a consistent increase of water deficit in the two basins. At the Ganges–Brahmaputra basin in all months (except July and August), water loss per year as indicated by the trend of TWSA is equivalent to the abnormally dry condition. As little change in SPI or SPEI is found, the water deficit is mainly a consequence of human impacts. In other words, water deficit caused by human activities every year is equivalent to water deficit in an abnormally dry situation, posing an inevitable anthropogenic trend on water availability. In the Markazi basin, the monthly trend in TWSA time series shows a deficit every year equivalent to a moderate drought (except February and March which corresponds to an abnormally dry condition). Thus, the anthropogenic impacts at the Markazi basin can be considered as a perpetual anthropogenic drought with accelerated severity. In general, anthropogenic drought refers to conditions in which human activities cause or intensify droughts^18^. In this particular case, the results show that human impacts mainly through overexploitation of groundwater has caused anthropogenic drought even when there is no precipitation deficit from a meteorological viewpoint. For this reason, a constant and alarming decline in TWS can be seen over time with water loss every year approximately equivalent to the deficit from a moderate drought.

### GRACE-based thresholds for drought recovery

**Figure 5:**
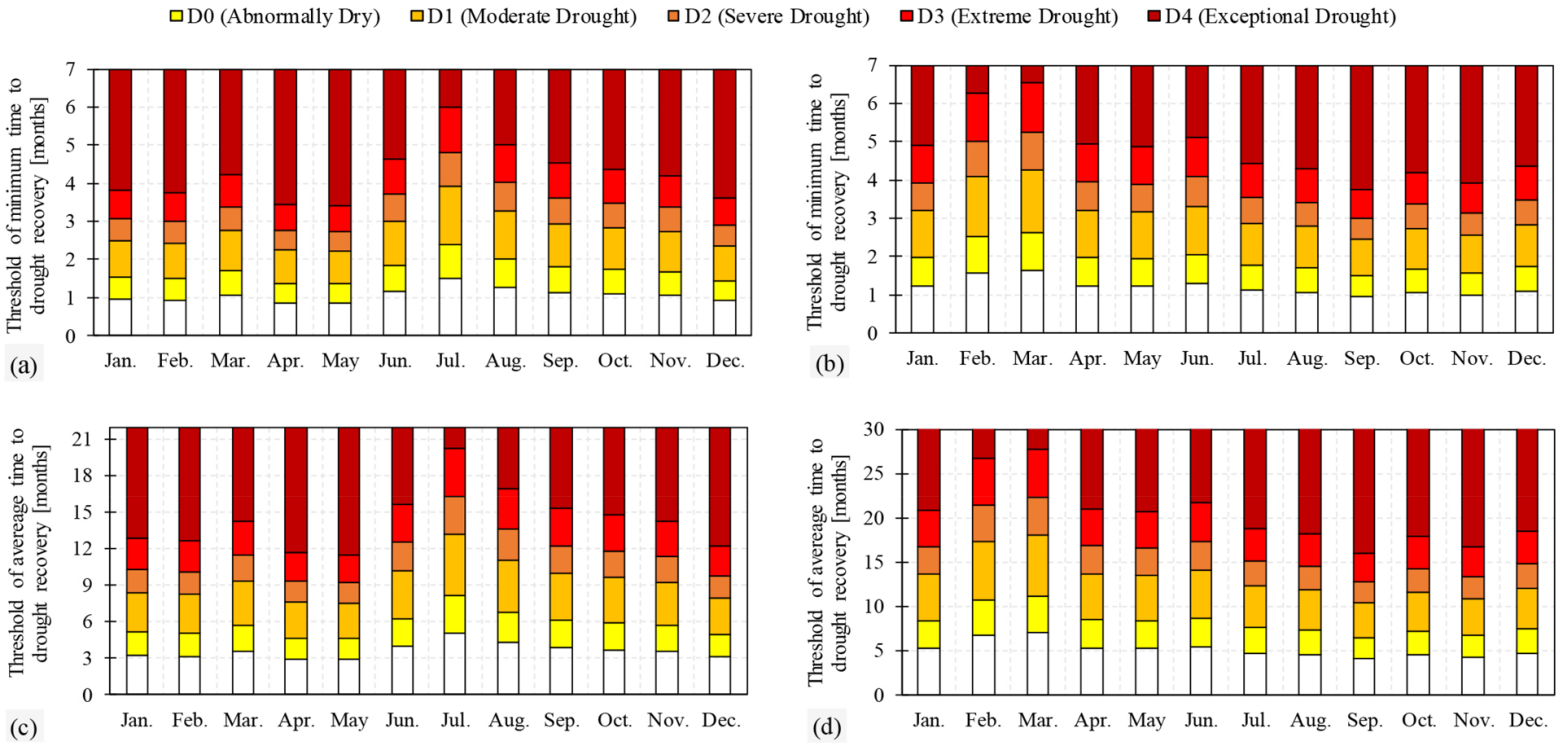
Thresholds of minimum and average time to drought recovery related to each drought category in each month for (**a**,**c**) the Ganges–Brahmaputra basin and (**b**,**d**) the Markazi basin.

Having estimates about water deficit (Fig. 4), we computed minimum and average time (months) to recover from each drought category in each month (Fig. 5)—see “Data and methods” section for details. Figure 5 shows how long it takes to recover from different deficits. For example, over the Ganges–Brahmaputra basin in January, for a recovery from an abnormally dry situation to normal situation, minimum 1–1.5 months and on average between 3.2 and 5.15 months are needed. As another example, to recover an exceptional drought corresponding to MTWSDI equal to − 2, minimum 3.8 and on average 12.8 months are required. For the Markazi basin, in January, an abnormally dry situation disappears minimum after 1.2–2 months and on average it will take between 5.2 and 8.3 months. In addition, for thresholds of moderate, severe, extreme and exceptional drought also minimum of 2, 3.2, 3.9 and 4.9 months and on average 8.3, 13.6, 16.7 and 20.8 months are required. Figure 5 shows the results for other months. It should be noted that absolute values of deficit thresholds and deficit changes in the Markazi basin are less than those in the Ganges–Brahmaputra basin (see “Data and methods” sections). Therefore, recovery times are not very different.

## Discussion

GRACE observations provide useful information for hydrological studies^62^. However, considering that its observations include imprints from both climatic variability and anthropogenic interventions, linking GRACE observations to meteorological variability or anthropogenic impact is not always straightforward. In this study, a novel approach was introduced for drought monitoring over basins with significant human water use using a signal decomposition technique. The proposed approach, named Modified Total Water Storage Deficit Index (MTWSDI), decomposes the original GRACE signal and uses the residuals of the signal instead for meteorological drought monitoring and assessment. This allows for separating any possible deterministic trend (e.g., due to unsustainable water use and long term meteorological trend) from relatively short term meteorological variability. We applied our approach for meteorological drought monitoring over the Ganges–Brahmaputra in India and Markazi basins in Iran. Our findings showed that MTWSDI exhibits consistency with meteorological drought indices (e.g., SPI and SPEI) in both basins. Comparing the GRACE-based indices with meteorological drought indices, the results indicated that the proposed MTWSDI outperformed the commonly used TWSDI. The new developed MTWSDI captured the meteorological droughts as indicated by the meteorological drought indices and reported in previous works^51, 63–67^. The TWSDI, however, showed significant downward trends that is likely caused by human-induced water deficit such as groundwater overexploitation. In line with previous studies, the results illustrated that GRACE observations may not be suitable to study short-term meteorological drought, while they are efficient for long-term meteorological and anthropogenic drought monitoring^41, 49^.

We, for the first time, have developed a threshold-based drought monitoring and recovery using GRACE observations, providing useful information about volume of deficits, and minimum and average time for drought recovery. This approach provides unique information for decision-makers for planning and management. Based on the developed thresholds, it is shown that water deficits caused by anthropogenic impacts every year as indicated by the trend of TWS of the Ganges–Brahmaputra basin and Markazi basins is almost equal to water deficit in an abnormally dry condition and a moderate drought, receptively. In other words, unsustainable human water use has led to a form of perpetual and accelerated anthropogenic drought. Continuation of this trend would deplete the basin and cause significant socio-economic challenges. As a final remark, the proposed approach may be applied in both heavy irrigated/managed basins as well as more natural systems.

## Data and methods

### Data availability

GRACE Release 06 Mascon Solutions^68^ produced by the Center for Space Research (CSR, https://www2.csr.utexas.edu/grace/RL06_mascons.html, Last accessed: 21 August 2019) with a 0.25° × 0.25° spatial resolution, were used in this study. While the CSR RL06 is available at a 0.25° scale, the native resolution is much courses and hence, the GRACE data is recommended for application to large basins (e.g., with an area larger than 200,000 km^2^^69^). The reason for using mascon solutions is that many studies, such as Scanlon et al.^70^, revealed that mascon solutions have advantages relative to the spherical harmonics solutions including less leakage error. Also among available mascon solutions, CSR mascon solutions can be used for hydrological applications without applying any gain factors or any post-processing^68, 71^. We calculated an area-weighted monthly TWSA time series of GRACE from April 2002 to March 2017, and applied a linear interpolation for filling monthly gaps in data with the same assumptions proposed by Solander et al.^38^. We assumed that the length of GRACE observations could be considered a representation of the long-term behaviour of TWSA over the basins. We acknowledge the limitations of this assumption. However, this is currently the longest satellite-based TWSA time series available. Also, for precipitation and potential evapotranspiration (PET), Climatic Research Unit (CRU) time series dataset^72^ version 4.03 (https://crudata.uea.ac.uk/cru/data/hrg/, Last accessed: 21 August 2019) were used between 1980 and 2018, respectively. Precipitation and potential evapotranspiration data had monthly temporal resolution and 0.5° × 0.5° spatial resolution. The root mean square (RSM) values of TWSA (plotted in Fig. 1) is calculated using Eq. (1) as follows:

1 $$RMS = \sqrt { \frac{1}{T} \left( {\sum\nolimits_{t = 1}^{T} {TWSA_{t}^{2} } } \right)}$$

where ${TWSA}_{t}$ is TWSA in period t and T is the length of TWSA time series.

### Drought indices

TWSDI used by Zhao et al.^41^ is a standardization of TWSA time series month-to-month as follows:

2 $${\text{TWSDI}}_{{{\text{i}} \cdot {\text{j}}}} = \frac{{{\text{TWSA}}_{{{\text{i}} \cdot {\text{j}}}} - \overline{{{\text{TWSA}}}} _{{\text{j}}} }}{{{\text{SD}}_{{{\text{TWSA}}_{{\text{j}}} }} }}$$

where ${\mathrm{TWSDI}}_{\mathrm{i}\cdot \mathrm{j}}$ is Total Water Storage Deficit Index for ith year and jth month, ${\mathrm{TWSA}}_{\mathrm{i}\cdot \mathrm{j}}$ is total water storage anomaly in ith year and jth month (mm), $\overline{{{\text{TWSA}}}} _{{\text{j}}}$ is average of TWSA for jth month (mm), and ${{\mathrm{SD}}_{\mathrm{TWSA}}}_{\mathrm{j}}$ is the standard deviation of TWSA for jth month (mm). As mentioned before, this TWSA in heavy irrigated basins is significantly affected by human water use, among other climatic factors. To address this issue, we used a signal decomposition technique. The GRACE signal consisted of four terms as follows^70^:

3 $$\mathrm{TWSA}=\mathrm{Trend}+\mathrm{Annual\;signal}+\mathrm{Semi-}\mathrm{annual\;signal}+\mathrm{Residuals}$$

where $\mathrm{TWSA}$ is total signal (mm), Trend is long-term behaviour of TWSA time series (mm), Annual and Semi-annual signals are annual and semiannual (i.e., representing seasonality) amplitudes of TWSA (mm), and Residuals (mm) are the difference between the TWSA and the sum of other three terms mentioned earlier. Except Residues, all other terms exhibit a deterministic behaviour and hence can be removed. TWSA trend can be removed using a linear trend line and Annual and Semi-annual signals can be extracted by fitting sines and/or cosines functions^70^. As a result, we can modify TWSDI as follows:

4 $${\text{MTWSDI}}_{{{\text{i}} \cdot {\text{j}}}} = \frac{{{\text{Residuals}}_{{{\text{i}} \cdot {\text{j}}}} - \overline{{{\text{Residuals}}}} _{{\text{j}}} }}{{{\text{SD}}_{{{\text{Residuals}}_{j} }} }}$$

where ${\mathrm{MTWSDI}}_{\mathrm{i}\cdot \mathrm{j}}$ is Modified Total Water Storage Deficit Index for ith year and jth month, ${\mathrm{Residuals}}_{\mathrm{i}\cdot \mathrm{j}}$ is remaining of total signal for ith year and jth month (mm), $\overline{{{\text{Residuals}}}} _{{\text{j}}}$ is average of Residuals related to jth month (mm), and ${\mathrm{SD}}_{{\mathrm{Residuals}}_{j}}$ is the standard deviation of Residuals for jth month.

Additionally, SPI and SPEI time series were calculated as a benchmark to evaluate GRACE-based drought indices. SPI and SPEI calculations were performed following recommendations of McKee et al.^73^ and Vicente-Serrano et al.^74^, respectively and considering suggestions of Stagge el al.^75^. SPI and SPEI were computed using precipitation and PET from CRU TS data. Table 1 presents categorization of the drought based on all indices used in this study.

### Deficit

We considered Eqs. (5) and (6) as the storage deficit:

5 $${\text{D}}_{{{\text{i}}.{\text{j}}}} = \left( {{\text{TWSA}}_{{{\text{i}}\cdot{\text{j}}}} - \overline{{{\text{TWSA}}}}_{{\text{j}}} } \right) \times Area \times 10^{ - 6}$$

6 $${\text{MD}}_{{{\text{i}}\cdot{\text{j}}}} = \left( {{\text{Residuals}}_{{{\text{i}}\cdot{\text{j}}}} - \overline{{{\text{Residuals}}}}_{{\text{j}}} } \right) \times Area \times 10^{ - 6}$$

where ${\mathrm{D}}_{\mathrm{i}.\mathrm{j}}$ and ${\mathrm{MD}}_{\mathrm{i}\cdot \mathrm{j}}$ are deficit and modified deficit (km^3^) for ith year and jth month, respectively, and $Area$ is the area of basin (km^2^).

### Drought severity

Drought Severity was considered as summation of deficits amounts during a given drought event. We computed the total severity during the drought event as follows:

7 $${\text{Severity }}_{n} = \overline{{\text{Deficit }}}_{n} \times {\text{Duration}}_{n}$$

where Severity is total severity within nth drought event (km^3^ months), $\overline{{\text{Deficit }}}_{n}$ is average of storage deficit (either D or MD) during nth drought event (km^3^) and Duration is number of months of nth event drought (months).

### Drought threshold

This study used the month-to-month drought index and deficit time series to define a deficit threshold for each drought category. We fitted liner equations between drought indices and their corresponding deficit for each month. Then, these equations were used to determine the deficit threshold based on upper bound of drought indices in each category (see Table 1) as follows:

8 $${\mathrm{Threshold}}_{j\cdot d}={\mathrm{a}}_{j}\times {UB}_{d}+{b}_{j}$$

where ${\mathrm{Threshold}}_{j\cdot d}$ is the deficit threshold in jth month for dth drought category, ${UB}_{d}$ is upper bound of drought index in dth drought category, and ${\mathrm{a}}_{j}$ and ${b}_{j}$ are the regression coefficient and intercept for jth month, respectively. Considering Eq. (4) and Eq. (6), ${\text{b}}_{{\text{j}}}$ would be equal to zero.

### Threshold of drought recovery

Based on time series of MD (or D), we can compute time series of deficit changes based on central difference approaches as:

9 $$\frac{d}{dt}\mathrm{DM}(\mathrm{t})=\frac{DM\left(t+\Delta t\right)-DM\left(t-\Delta t\right)}{2\Delta t}$$

where $\frac{d}{ dt}\mathrm{DM}(\mathrm{t})$ is deficit change in tth time step, and for the monthly evolution $\Delta t$ is equal to one month. This study used the approach introduced by Thomas et al.^42^ to determine drought recovery based on fitting empirical (Kaplan–Meier) cumulative distribution (eCDF) on $\frac{d}{ dt}\mathrm{DM}(\mathrm{t})$ time series. The values corresponding to probability of 0.95 and 0.68, based on fitted eCDF, represent the maximum and average positive change of deficits^42^. Finally, we divided drought deficit thresholds by maximum and average rate of deficit changes to calculate thresholds of minimum and average time to recovery, respectively.

### Separating monitoring metrological drought from anthropogenic impact

We have used the residuals and trend of TWSA to quantity metrological drought and anthropogenic impact, respectively. We acknowledge that this is a proxy approach and it is subject to uncertainty, especially in areas where there is a significant evidence of change in meteorological droughts. To justify our assumption, we have analyzed the time series of precipitation and potential evapotranspiration as the main variables that control water availability and meteorological droughts in a natural basin. During the period of GRACE observations (2002–2017), at both study areas, the datasets do not exhibit any statistically significant trend based on a Mann Kendall test at the 0.01 significance level (99% confidence level). Therefore, it is reasonable to assume that the decreasing trend in TWSA is related to human activity not meteorological changes. In such a situation, the possible trends in time series should be removed for monitoring meteorological drought. Further, removing the seasonality (climatology) is a commonly used in drought indices not only GRACE-based drought indices but also other standardized drought indices. After removing the trend and seasonality, the residuals remain that exhibit random patterns consistent with meteorological variability (but significantly different than permanent signals such as increased human water use over time). Therefore, de-trended and de-seasonalized GRACE TWSA can be used as proxy to extract the climatic drought signals^76^. In the next step, we calculated the volume of water that the two basins lost every year due to the downward trend. Then, based on the volume of water lost in each month due to human water use (based on the trend data) relative to the threshold of deficit for each drought category in each month (see Fig. 4), we separated meteorological droughts and anthropogenic impacts. It should be noted that another approach for separating meteorological droughts from anthropogenic droughts is using ancillary data like outputs of land surface models (LSMs) or global hydrological models (GHMs). This approach would be preferred upon availability of reliable and calibrated model simulations. However, most of the existing global models and even local land-surface models are uncalibrated^77^ and many of them do not simulate groundwater at all^78^. Therefore, using their outputs for separating meteorological and anthropogenic factors are subject to large uncertainty.
